# SMS messages increase adherence to rapid diagnostic test results among malaria patients: results from a pilot study in Nigeria

**DOI:** 10.1186/1475-2875-13-69

**Published:** 2014-02-25

**Authors:** Sepideh Modrek, Eric Schatzkin, Anna De La Cruz, Chinwoke Isiguzo, Ernest Nwokolo, Jennifer Anyanti, Chinazo Ujuju, Dominic Montagu, Jenny Liu

**Affiliations:** 1 Stanford University, School of Medicine, General Medical Disciplines, 1070 Arastradero Rd, Palo Alto, CA, USA; 2 University of California, San Francisco, Global Health Group, 50 Beale St, San Francisco, CA, USA; 3 Society for Family Health, No 8 Port Harcourt Crescent, Area 11, Garki, Abuja, Nigeria

**Keywords:** Malaria, Nigeria, Adherence to rapid diagnostic test for malaria, SMS intervention

## Abstract

**Background:**

The World Health Organization now recommends parasitological confirmation for malaria case management. Rapid diagnostic tests (RDTs) for malaria are an accurate and simple diagnostic to confirm parasite presence in blood. However, where they have been deployed, adherence to RDT results has been poor, especially when the test result is negative. Few studies have examined adherence to RDTs distributed or purchased through the private sector.

**Methods:**

The Rapid Examination of Malaria and Evaluation of Diagnostic Information (REMEDI) study assessed the acceptability of and adherence to RDT results for patients seeking care from private sector drug retailers in two cities in Oyo State in south-west Nigeria. In total, 465 adult participants were enrolled upon exit from a participating drug shop having purchased anti-malaria drugs for themselves. Participants were given a free RDT and the appropriate treatment advice based on their RDT result. Short Message Service (SMS) text messages reiterating the treatment advice were sent to a randomly selected half of the participants one day after being tested. Participants were contacted via phone four days after the RDT was conducted to assess adherence to the RDT information and treatment advice.

**Results:**

Adherence to RDT results was 14.3 percentage points (P-val <0.001) higher in the treatment group who were sent the SMS. The higher adherence in the treatment group was robust to several specification tests and the estimated difference in adherence ranged from 9.7 to 16.1 percentage points. Further, the higher adherence to the treatment advice was specific to the treatment advice for anti-malarial drugs and not other drugs purchased to treat malaria symptoms in the RDT-negative participants who bought both anti-malarial and symptom drugs. There was no difference in adherence for the RDT-positive participants who were sent the SMS.

**Conclusions:**

SMS text messages substantially increased adherence to RDT results for patients seeking care for malaria from privately owned drug retailers in Nigeria and may be a simple and cost-effective means for boosting adherence to RDT results if and when RDTs are introduced as a commercial retail product.

## Background

Although recent World Health Organization guidelines for malaria management in endemic countries recommend that treatment should be reserved for confirmed malaria cases [1], presumptive diagnosis of malaria and subsequent over-administration of anti-malarial drugs remain the norm [2]. With the recent improvements in the accuracy of rapid diagnostic tests (RDTs) for malaria, there is an opportunity to improve the quality of diagnosis and treatment. RDTs are antigen-based tests that detect parasite-specific antibodies or antigens in a drop of blood within 15 minutes. RDTs are easy to use and have similar or superior specificity and sensitivity to microscopy, the previous gold-standard diagnostic [3,4]. The relative simplicity of RDTs enables their use by those with minimal medical training [5] and their low cost make them a potentially cost-effective intervention for malaria management in low resources settings [6].

There has been a rapid increase in the availability of RDTs throughout many sub-Saharan African countries [7]. However, patient adherence to test results has been poor, especially when the test results are negative [8,9]. When RDT-negative patients disregard RDT results, the potential of RDTs to increase the cost-effectiveness of malaria treatment is undermined because 1) savings from unnecessary treatments are not realized, and 2) over-administration of first-line anti-malarial drugs may fuel parasite resistance and/or diminished efficacy over time.

Strategies to improve adherence to RDTs have been attempted in countries where the public sector has implemented strict case management guidelines. In Senegal, where artemisinin-based combination therapy (ACT) is only given with a positive RDT result, the number of ACT prescriptions now matches malaria prevalence [10]. However, such strategies are unlikely to be successful in countries where the majority of health services are provided through the private sector, such as in Nigeria [11]. In these countries, alternative strategies to reinforce treatment adherence to test results may be needed in lieu of strong regulatory control.

A series of recent studies have shown that reminders sent via short message service (SMS) can ‘nudge’ people to increase adherence to a variety of health related behaviours, including applying sunscreen [12], remembering to take diabetes drugs [13], following HIV antiretroviral therapy treatment regimens [14], and adhering to asthma treatment [15]. For RDT use in malaria case management, a ‘nudge’ may help people adhere to RDT results among those who are hesitant to follow through because of insufficient previous experience. Reinforcement of the appropriate health behaviour indicated by the RDT may be particularly valuable when the test result conflicts with prior expectations of having malaria. While there is some consensus that SMS-based reminder interventions may be well-suited to developing countries [16,17], there has not been any work to date to examine the extent to which SMS messages can be leveraged to increase adherence to RDT results.

This study evaluates a pilot SMS-based intervention included in the Rapid Examination of Malaria and Evaluation of Diagnostic Information (REMEDI) study conducted in Oyo State in south-west Nigeria. The goal of the REMEDI study was to evaluate the acceptability of RDTs among people seeking malaria drugs from private sector drug retailers (detailed below in study design section). As a part of the study, half of the enrolled participants were randomly assigned to receive an SMS message, which 1) reiterated the participant’s RDT result and corresponding treatment advice, and 2) provided a platform whereby participants could send their questions to an advice nurse via SMS.

## Methods

### Study design

The REMEDI SMS pilot was a double-blinded, parallel-group study conducted in Oyo state in south-west Nigeria, in and around the cities of Ibadan and Ogbomosho. Ibadan is a large urban center, while Ogbomosho is mainly peri-urban. The main goal of the REMEDI study was to examine the acceptability of RDTs among customers of private sector retail outlets. Privately owned pharmacies and proprietary and patent medicine vendors (PPMVs) were initially randomly selected from the enumerated sites within four local government areas and enrolled into the study. Two weeks after the beginning of the study, the site selection was modified to exclude small drug retailers whose main business was not medicinal sales; these PPMVs were replaced with other PPMVs in the adjacent local government areas.

At each retail site, adult customers exiting the shop were approached, screened for eligibility, and asked to complete a short survey. In brief, the protocol entailed 1) enrolling non-pregnant adults outside of a pharmacy/drug shop who stated and demonstrated that they had just purchased malaria treatment for him/herself; 2) offering and conducting an RDT performed by a trained nurse; 3) taking a detailed survey and inventory of drugs purchased; 4) discussing the test result with the patient; and 5) providing appropriate treatment advice based on the test result at the end of the survey. If the participant’s RDT was positive, the patient was told that the positive result indicates the presence of malaria and was instructed to take a course of ACT, that was provided for free. If the participant’s RDT test was negative, the patient was told that the negative result indicates the absence of malaria and that anti-malarial drugs would not be needed, but could be saved for use at a later time. The nurse informed participants that they could still take other medications they purchased for other conditions or to treat symptoms.

Regardless of the test result, all participants were referred to local clinics and hospitals where they could seek care if their condition was not malaria or if their illness became worse. All participants were also given an informational card that outlined treatment steps based on their test result. Lastly, participants were asked for their cell phone contact information and told to expect a short 5–10 minute phone call in four days to check on the status of their illness, during which they would be compensated with 100 Naira (US$0.63) in phone credits for taking the call.

In the follow-up phone survey four days after the initial encounter, participants were asked which of the purchased drugs they had used. Responses to this question were used as a measure of adherence to the RDT information and treatment advice.

### SMS intervention protocol and complications

SMS messages were sent to randomly selected participants one day after the initial encounter. The same message was sent to each participant once in English and once in Yoruba (the dominant local ethnic group). The content of the message repeated the advice given by the nurse the previous day at the time of testing and included a final line stating that patient could reply to the nurse via text if they need additional information.

Two text messages were sent based on the participant’s RDT, one in English and a second in Yoruba. RDT positive participants were sent the following message in English, “Yesterday, you tested POSITIVE for malaria. Take LUMARTEM. If questions, please reply or TEXT. A nurse will call you soon.” A second message was sent separately in Yoruba, “Lanaa, iwadii fi han pe E NI IBA. E maa lo ACT yin bo se ye. Te e ba ni ibeere, e fesi ateranse yii, noosi wa yoo si daa yin lohun logan.” RDT negative participants were sent the following message in English, “Yesterday, you tested NEGATIVE for malaria. Malaria drugs NOT needed. If questions, please reply or TEXT. A nurse will call you soon.” Again a second message was sent separately in Yoruba, “Lanaa, iwadii wa han pe E O NI IBA. Eyi fi han pe e o nilo oogun iba. Te e ba ni ibeere, e fesi ateranse yii, noosi wa yoo si daa yin lohun logan.”

To ensure that none of the survey staff would know who were chosen to receive the SMS, the study manager, who did not have any interaction with participants, randomly assigned surveys into the treatment group after the surveys were returned to the study office each day. The protocol for treatment assignment entailed assigning every other survey to the SMS treatment group on the day of the baseline survey. However, due to disruptions in electricity and communications channels with the study site in Ogbomosho, surveys collected in Ogbomosho were not returned to the study office in time for assignment to treatment/control group the same day. On days when disruptions prevented the study manager from following assignment protocol, treatment assignment was slightly altered. Additional surveys from Ibadan were randomly assigned to the treatment group to compensate for fewer treatment assignments from Ogbomosho. During these periods of delay in transmissions from Ogbomosho, one additional Ibadan-based survey was selected to receive an SMS for every two surveys collected in Ogbomosho. These “off-protocol” treatment assignments are taken into account in the statistical analyses of the data.

### Participants

**Figure 1:**
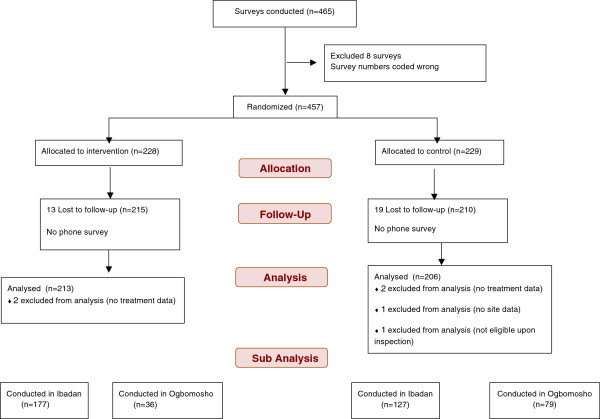
Data exclusion flow chart.

Of 465 adults enrolled adults, all of whom completed the baseline survey; 32 participants were not reached for follow-up. An additional eight surveys had duplicated survey numbers, so these observations were dropped, as it was impossible to tell which entry was correct. With these exclusions, 425 participants remain who were reached in the follow-up phone survey. Only 419 observations are analysed because four surveys did not have drug information data, one was missing site information and one was ineligible upon further inspection. In total, 213 participants were sent the SMS reminder one day after their RDT was conducted at the phone number they provided. Figure 1 presents a flow chart outlining the surveys included for analysis.

### Statistical analyses

Descriptive analyses present sample characteristics, the randomization of participants, and the distribution of variables between treatment and control groups. The main analysis examines whether participants adhered to the recommended treatment course based on their RDT result. Specifically, participants are considered to have “followed treatment advice” if (1) they were RDT-negative and did not take any of the anti-malarial medications purchased or (2) if they were RDT-positive and took ACT. This binary outcome is predicted using logistic regression to compare the SMS treatment group to those who were not sent an SMS. Standard errors are clustered by retail site where the surveys were conducted.

The primary analysis estimates the “intent to treat” effect. Mean differences across treatment and control groups, odds ratios (risk ratio), and risk differences (marginal effects) are reported. Four model specifications are employed:

Model 1

$$\mathrm{Log}\left(\mathrm{Adhere}/\left(1-\mathrm{Adhere}\right)\right)=a+b\left(\mathrm{SMS}\right)+e$$

Model 2

$$\begin{array}{l} \mathrm{Log}\left(\mathrm{Adhere}/\left(1-\mathrm{Adhere}\right)\right)=a+b\left(\mathrm{SMS}\right)\\ \;\;\;\;\;\;+g\left(\mathrm{SMS}*\mathrm{Ibadan}\right)\\ \;\;\;\;\;\;+\pi \left(\mathrm{Ibadan}\right)+e \end{array}$$

Model 3

$$\begin{array}{l} \mathrm{Log}\left(\mathrm{Adhere}/\left(1-\mathrm{Adhere}\right)\right)=a+b\left(\mathrm{SMS}\right)\\ \;\;\;\;\;\;+g\left(\mathrm{SMS}*\mathrm{Ibadan}\right)\\ \;\;\;\;\;\;+\pi \left(\mathrm{Ibadan}\right)\\ \;\;\;\;\;\;+h\left(\mathrm{SMS}*\mathrm{Off}-\mathrm{protocol}\right)\\ \;\;\;\;\;\;+\mu \left(\mathrm{Off}-\mathrm{protocol}\right)+e \end{array}$$

Model 4

$$\begin{array}{l} \mathrm{Log}\left(\mathrm{Adhere}/\left(1-\mathrm{Adhere}\right)\right)=a+b\left(\mathrm{SMS}\right)\\ \;\;\;\;\;\;+g\left(\mathrm{SMS}*\mathrm{Ibadan}\right)\\ \;\;\;\;\;\;+\pi \left(\mathrm{Ibadan}\right)\\ \;\;\;\;\;\;+h\left(\mathrm{SMS}*\mathrm{Off}-\mathrm{protocol}\right)\\ \;\;\;\;\;\;+\mu \left(\mathrm{Off}-\mathrm{protocol}\right)\\ \;\;\;\;\;\;+\rho \left(\mathrm{Unbalance}\;\mathrm{variables}\right)\\ \;\;\;\;\;\;+e \end{array}$$

Model 1 compares the difference in mean adherence between the treatment and the control group. To account for potential differences in the effectiveness of the SMS intervention between Ogbomosho and Ibadan, Model 2 adds an indicator variable for each city and an interaction term between the treatment and the city in which participants were surveyed. In Model 3, an interaction term for SMS treatment assignment on off-protocol days and an indicator for off-protocol assignment are added to examine potential differences due to disruptions experienced in Ogbomosho. Lastly, Model 4 adds controls for observable sociodemographic variables that are unbalanced across groups to further control for potential differences between treatment and control individuals.

Two additional outcomes are analysed. A secondary analysis examines the effect of the SMS intervention on those who report actually seeing the SMS, or the “treatment on the treated effect.” This analysis excludes 39 participants who were sent an SMS, but who reported that they did not see it. Lastly, to determine whether the SMS influenced only anti-malarial drug adherence versus other non-malarial drugs purchased, the same regression analyses are used to predict the likelihood of taking malaria and non-malarial drugs among a subset of RDT-negative participants who bought both types of drugs. While the SMS message only provided advice on the appropriate use of anti-malarial drugs, it is unclear whether this advice also affected participants’ usage of non-malarial drugs that are beneficial for treatment of symptoms. This analysis captures the specificity of the response to the content of the SMS message by examining the relationship between receiving the SMS message and usage of both anti-malarial and symptom drugs. For these additional outcomes group differences, odds ratios (risk ratio), and risk differences (marginal effects) are reported, as well as results for regression models 1 and 3.

### Ethical considerations

The University of California, San Francisco’s institutional review board and Nigeria’s National Health Research Ethics Committee approved the REMEDI study protocol. Surveyors obtained written consent from all study participants.

## Results

Table 1 presents sample characteristics for participants across treatment and control groups. The intervention and control groups are significantly different in terms of literacy and having cable TV, a motorcycle, a bank account, a flush toilet, and RDT-positive prevalence. Once the city in which the survey was conducted is controlled for, only having a bank account (88.7% vs. 74.8%) and not being able to read or write (98.1% vs. 91.7%) remain significantly different. Hence, these unbalanced variables are controlled for in subsequent regression model specifications.

**Table 1:** Summary statistic for treatment and control samples

	**SMS sent (N = 213)**		**SMS not sent (N = 206)**	
	N	Mean or %	N	Mean or %
**Sites**				
PPMV^&^	81	38.0%	109	52.9%
Ogbomosho^&^	36	16.9%	79	38.3%
**Demographics**				
Male	109	51.2%	102	50.0%
Age		40.7		37.3
Married	157	73.7%	131	63.9%
**Literacy status**				
Can not read or write^*^^&^	209	98.1%	189	91.7%
**Education level**				
Less than primary	14	6.6%	16	7.8%
Completed primary	27	12.7%	29	14.1%
Completed secondary	88	41.5%	76	37.1%
More than secondary	83	39.2%	84	41.0%
**Assets/infrastructure**				
Electricity	204	95.8%	196	95.1%
Radio	199	93.4%	189	91.7%
TV	193	90.6%	188	91.3%
Refrigerator	144	67.6%	129	62.6%
Cable TV^&^	114	53.8%	90	43.7%
Generator	152	71.4%	144	70.6%
AC	36	16.9%	29	14.1%
Computer	75	35.4%	78	38.0%
Electric iron	195	91.5%	179	87.3%
Fan	194	91.1%	189	92.2%
Motorcycle/scooter^&^	36	16.9%	58	28.3%
Car	102	47.9%	91	44.2%
Bank account^*^^&^	189	88.7%	154	74.8%
Flush toilet in house^&^	171	80.3%	144	69.9%
Kerosene to cook	116	54.5%	126	61.2%
Concrete floors	139	65.9%	126	62.1%
**RDT prevalence**				
RDT positive&	12	5.6%	4	1.9%

^&^Variables are statistically different across treatment and control groups.

^*^Variables remain statistically different across treatment and control groups even within city (Ibadan/Ogbomosho0.)

Table 2 shows estimates of the intent to treat analysis. A simple comparison of means shows that 79.8% of participants who were sent an SMS adhered to the treatment advice compared to only 65.5% who were not sent an SMS. This translates into a 14.3 percentage point (95% CI: 6.68-21.9 percentage points) increase in adherence, with an odds (risk) ratio of 2.08 (95% CI: 1.44-3.01). When controlling for and allowing for different treatment effect by location in row 2, the estimate reduces to a 10.1 percentage point difference (95% CI: 3.66-16.6 percentage points) between the treatment and control groups, with an odds (risk) ratio of 1.70 (95% CI: 1.20-2.42). Further accounting for the influence of assigning treatment to SMS reminders off-protocol in row 3 and controlling for unbalanced baseline asset variables in row 4 returns treatment effect estimates similar to the unadjusted estimates in row 1.

**Table 2:** Logistic regression results for the intent to treat effect of SMS reminders on adherence to treatment advice

**Primary outcome**	**Percentage (N)**		**SMS**	**SMS**
	**SMS sent (213)**	**SMS not sent (206)**	**SMS risk ratio (95% CI)**	**Risk difference (95% CI)**
1 Followed treatment advice	79.8% (170)	65.5% (135)	2.08 (1.44–3.01)	14.3% (6.68%–21.9%)
2 Followed treatment advice*			1.70 (1.20–2.42)	10.1% (3.66%–16.6%)
3 Followed treatment advice**			2.19 (1.50–3.18)	14.8% (8.14%–21.4%)
4 Followed treatment advice***			2.13 (1.45–3.12)	14.2% (7.33%–21.1%)

N = 419.

*Controls for city survey was conducted (Ibadan/Ogbomosho) and treatment*city survey was conducted (Model 2).

**Controls for city survey was conducted (Ibadan/Ogbomosho), treatment*city survey was conducted, whether assignment to the treatment/control group was made off-protocol, and treatment* whether survey assignment to the treatment/control group was made off-protocol (Model 3).

***Controls for city survey was conducted (Ibadan/Ogbomosho), treatment*city survey was conducted, whether assignment to the treatment/control group was made off-protocol, and treatment* whether survey assignment to the treatment/control group was made off-protocol, an indicator variable for surveys conducted at PPMVs, an indicator variable for participant who had a bank account, and an indicator variable for participant could not read or write (Model 4).

Standard errors are clustered by retail site.

In Table 3, estimates of the treatment effect on the treated excluding 39 participants who reported that they did not see the SMS are presented. Adherence to the treatment advice in the SMS group increases to 81.6%. Unconditional means in row 1 show that adherence was higher in the SMS group by 16.1 percentage points (95% CI: 8.89-23.2 percentage points). The SMS group had a higher likelihood of adhering to treatment advise with an odds (risk) ratio of 2.33 (95% CI: 1.62-3.36). In specifications where differences by city and/or issues of off-protocol assignment were accounted for, the estimated treatment effect on the treated ranges from 11.3 percentage points (95% CI: 3.76-18.9 percentage points) to 15.5 percentage points (95% CI: 7.86- 23.2 percentage points).

**Table 3:** Logistic regression estimates of the treatment on treated effect of SMS reminders on adherence to treatment advice

**Primary outcome**	**Percentage (N)**		**SMS**	**SMS**
	**Received SMS (n = 174)**	**SMS not sent (n = 206)**	**SMS risk ratio (95% CI)**	**Risk difference (95% CI)**
1 Followed treatment advice	81.6% (142)	65.5% (135)	2.33 (1.62–3.36)	16.1% (8.89%–23.2%)
2 Followed treatment advice*			1.84 (1.19–2.85)	11.3% (3.76%–18.9%)
3 Followed treatment advice**			2.34 (1.46–3.74)	15.5% (7.86%–23.2%)
4 Followed treatment advice***			1.68 (1.08–2.61)	9.68% (1.84%–17.5%)

Exclude 39 observations who were sent a text message but who reported not seeing the text message in the follow-up survey, N = 380.

*Controls for city survey was conducted (Ibadan/Ogbomosho) and treatment* city survey was conducted (Model 2).

**Controls for city survey was conducted (Ibadan/Ogbomosho), treatment* city survey was conducted, whether assignment to the treatment/control group was made off-protocol, and treatment* whether survey assignment to the treatment/control group was made off-protocol (Model 3).

***Controls for city survey was conducted (Ibadan/Ogbomosho), treatment* city survey was conducted, whether assignment to the treatment/control group was made off-protocol, and treatment* whether survey assignment to the treatment/control group was made off-protocol, an indicator variable for surveys conducted at PPMVs, an indicator variable for participant who had a bank account, and an indicator variable for participant could not read or write (Model 4).

Standard errors are clustered by retail site.

In Table 4, only the subset of RDT-negative participants who bought both symptom drugs and anti-malarial drugs are analysed. Of these 240 RDT-negative participants, 23.1% who were sent an SMS took their purchased anti-malarial drugs compared to 41.5% who were not sent an SMS. In other words, significantly fewer participants—18.4 percentage points (95% CI: −30.1 to −6.7 percentage points)—who were sent an SMS took the unnecessary anti-malarial drugs. For comparison, 73.5% of participants who were sent an SMS took their symptomatic drugs compared to 80.5% were not sent an SMS, a difference of 7 percentage points that was not statistically significant.

**Table 4:** Logistic regression results for the effect of SMS reminders on malaria and non-malaria drug-taking

**Medications taken**	**Percentage (N)**		**SMS**	**SMS**
	**SMS sent (n = 117)**	**SMS not sent (n = 123)**	**SMS risk ratio (95% CI)**	**Risk difference (95% CI)**
1 Took anti-malarial	23.1% (27)	41.5% (51)	0.42 (0.25–0.72)	−18.4% (−30.1% to −6.7%)
2 Took anti-malarial**			0.37 (0.20–0.69)	−20.6% (−32.5% to −8.8%)
3 Took symptom drug	73.5% (86)	80.5% (99)	0.67 (0.37–1.20)	−7.0% (−17.3% to 3.31%)
4 Took symptom drug**			0.89 (0.42–1.90)	−2.1% (−15.2% to 11.1%)

Sample includes RDT negative participants who bought both an antimalarial drug as well as drugs to treat their symptoms, N = 240.

**Controls for city survey was conducted (Ibadan/Ogbomosho), treatment* city survey was conducted, whether assignment to the treatment/control group was made off-protocol, and treatment* whether survey assignment to the treatment/control group was made off-protocol (Model 3).

Standard errors are clustered by retail site.

Table 5 presents the patterns of anti-malarial drug use for RDT-positive participants. There were only 16 RDT-positive participants so regression analyses are not conducted. Of these 16 RDT-positive participants, 75% took anti-malarial drugs regardless of whether they were sent an SMS or not. All RDT-positive participants who bought symptom drugs reported that they took these drugs.

**Table 5:** Medication use for RDT positive participants

**Medication use**	**Percentage (N)**	
	**SMS sent (n = 12)**	**SMS not sent (n = 4)**
Took anti-malarial	75% (9)	75% (3)

N = 16.

## Discussion

This study demonstrated that a simple SMS text message substantially increased adherence to RDT results. Compared to those who were not sent a reminder message, the probability of adhering to the correct malaria treatment advice increases by 10–15 percentage points among those who were sent the reminder. Previous estimates of the magnitude in the effect size of SMS interventions to increase adherence to drug treatments range from 8-17% [12-14,18] —the estimates in this study are very much in line with those studies. Together, these studies suggest that for many health-related behaviours, individuals may benefit from additional reinforcement to help them to follow through with intended or prescribed behaviours.

Unlike previous SMS intervention studies in which the message often persuaded the participant to do something that the individual already stated that s/he wanted to do (e.g. use sunscreen, take medication, or save money), in this study the message reinforces a previously given medical advice, which likely contradicted the planned behaviour of the participants. Indeed, the vast majority of the participants tested were found to have negative results (96%). These individuals, having just purchased treatment medicines, were essentially asked to change behaviour and **not** do something they usually would do—take an anti-malarial drug—to treat a suspected episode of malaria. This suggests that reminder messages can help individuals break from their default behaviours in addition to helping them follow through with intended behaviours.

To further understand how the SMS intervention may have influenced participants’ choices, participants in the treatment group were asked to describe which aspect of the SMS message they found helpful. The majority of respondents (52%) said that the SMS reminded them of their test result, while another 30% said that it reminded them of the correct treatment course. Only about 10% mentioned the usefulness of having a link to an advice nurse and only 10 participants actually contacted the advice nurse for consultation regarding unresolved febrile symptoms or to confirm that they should not take the anti-malarial medication. Hence, it appears that the reinforcement of the medical advice reiterated by the SMS was the most helpful to participants in choosing which drugs to take for their condition.

Given the ubiquity of cell phones throughout Nigeria and the relatively low cost of sending an SMS compared to the cost of anti-malaria drugs, the SMS intervention is cost-effective from a societal point of view. The average cost of the anti-malaria treatment course purchased within the study sample was 350 Naira (US$2.50). This cost reflects only the retail price of treatments and does not include the large-scale international subsidy for ACT, which is about $4.00 a course. Based on the results of this study, a similar SMS intervention would save $0.25 per episode in direct treatment costs alone. If the proportion of participants who bought ACT and international subsidy were taken into consideration, then the cost savings would average $0.43 per episode. Rough estimates of the cost of sending an SMS manually is about 10 cents including labour and service costs, suggesting that the SMS intervention is a cost-saving one. If the SMS messages were automated the cost be would even lower.

This study provides further evidence that reminding or nudging people can alter health-related behaviours. However, these results should be interpreted in light of several caveats. For various reasons, randomization was not perfectly balanced, although application of regression controls helped to adjust for these factors. Because the pilot study was conducted in primarily urban areas, this sample is unlikely to be representative of the state or of the country. Results reflect a wealthier and more educated population and health behaviours may differ, including responses to SMS messages, for poorer populations located in rural areas. An expanded version of this study is currently underway to assess the extent to which SMSs increase RDT adherence in other states, in areas with higher entomological inoculation rates, and in more representative populations. Further, because adherence to treatment advice is a self-reported measure, some reporting bias may be present if the SMS message also prompted individuals to self-report “better” outcomes. To the extent that the SMS increased reporting bias, the intervention effect size could be over-estimated. However, because results for malaria drugs and non-malaria drugs showed differential drug-taking behaviour, such self-reporting bias may be minimal. Finally, the SMS contained the exact same information that was provided by the advice nurse. For this reason, the SMS may have served as a general reminder of the testing experience, and in the present study the effect of a general reminder from the effect of the precise message contained within the SMS could not be differentiated. Future interventions would need to test different wording of the message to see which word choices increase adherence the most.

Currently, innovations in integrating RDTs into malaria case management in the private sector are well underway. Though this pilot study was limited to customers seeking care for malaria in the private sector healthcare market in Nigeria, RDT roll out is likely to expand in all sectors, and the results of this pilot study could have broader implications. The results suggest that SMS may be a tool to increase adherence to RDT results and could be used by many different health care practitioners (such as community health workers or hospital nurses) to support patients in following appropriate treatment advice. Coupling the RDT roll out with reinforcement messaging could help bring ACT use in line with actual malaria cases.

## Abbreviations

ACT: Artemisinin-based combination therapy; CI: Confidence interval; PPMV: Proprietary and patent medicine vendors; RDT: Rapid diagnostic tests for malaria; REMEDI: Rapid examination of malaria and evaluation of diagnostic information study; SMS: Short message service; WHO: World health organization.

## Competing interests

The authors declare they have no competing interests.

## Authors’ contributions

SM & JL conceived of the intervention. SM, JL, ES & AD wrote questionnaire, operations manuals, and trained staff for REMEDI study. ES implemented and oversaw SMS intervention. CI, EN, JA, CU & DM facilitated the intervention through funding and infrastructure of wider REMEDI study. SM did analysis. SM structured and wrote the manuscript. JL ES & AD participated in the presentation of results and contributed to writing the manuscript. All authors read and approved the final manuscript.
